# Attentional Processing of Disgust and Fear and Its Relationship With Contamination-Based Obsessive–Compulsive Symptoms: Stronger Response Urgency to Disgusting Stimuli in Disgust-Prone Individuals

**DOI:** 10.3389/fpsyt.2021.596557

**Published:** 2021-06-07

**Authors:** Jakob Fink-Lamotte, Andreas Widmann, Konstantin Sering, Erich Schröger, Cornelia Exner

**Affiliations:** ^1^University of Leipzig, Clinical Psychology and Psychotherapy, Leipzig, Germany; ^2^University of Leipzig, Cognitive and Biological Psychology, Leipzig, Germany; ^3^Leibniz Institute for Neurobiology, Magdeburg, Germany; ^4^University of Tuebingen, Quantitative Linguistics, Tübingen, Germany

**Keywords:** attention, facial expression, psychophysiology, obsessive-compulsive disorder, disgust, fear

## Abstract

Disgust has recently been characterized as a low-urgency emotion, particularly compared to fear. The aim of the present study is to clarify whether behavioral inhibition during disgust engagement is characteristic of a low-urgency emotion and thus indicates self-imposed attentional avoidance in comparison to fear. Therefore, 54 healthy participants performed an emotional go/no-go task with disgust- and fear-relevant as well as neutral pictures. Furthermore, heart rate activity and facial muscle activity on the fear-specific m. corrugator supercilli and the disgust-specific m. levator labii were assessed. The results partially support the temporal urgency hypothesis of disgust. The emotion conditions significantly differed in emotional engagement and in the facial muscle activity of the m. levator labii as expected. However, contrary to our expectations, no differences between the emotion conditions regarding behavioral inhibition as well as heart rate change could be found. Furthermore, individuals with a higher-trait disgust proneness showed faster reactions and higher activity of the m. levator labii in response to disgust stimuli. The results show that different trait levels influence attentional engagement and physiological parameters but have only a small effect on behavioral inhibition.

## Introduction

On the one hand, there is a long tradition of emotion research that assumes distinct emotion categories (1); on the other hand, there are more recent approaches that show clear evidence for the dimensional nature of emotions (2). Several findings show that both fear and disgust are maintaining emotions of contamination-related obsessive–compulsive disorder (OCD) (3). When the two emotions are altered by psychotherapeutic intervention exposure with response prevention, habituation and extinction problems are evident only for disgust experience compared to fear experience (4–8). To better understand this difference, we decided to follow the approach of distinct emotions for this study. This difference between the two emotions may be due to attentional avoidance and cognitive deficits of inhibition (9). The aim of the present study is to use a multimethod approach to understand more precisely to what extent disgust and fear differ in terms of behavioral inhibition in the context of subclinical contamination expressions.

Disgust is characterized as part of a disease avoidance system (10). At the behavioral level, disgust leads to freezing, avoidance, and withdrawal (11). On the other hand, fear is part of the defense and protection system that protects against objective, acute threat (12) and leads to fighting, fleeing, and freezing behavior. The differences between disgust and fear have recently been characterized in relation to the temporal urgency of the reaction (13–15). Knowles et al. (16) wrote in their functional perspective on attentional avoidance to disgust that fear is a high-urgency emotion, whereas disgust is a low-urgency emotion. Individuals who feel disgusted—unlike those who feel anxious—do not have to continuously observe the stimuli because there is no acute danger of body harm (17, 18). However, several EEG studies have shown that, particularly in early processing (P2 component, 200 ms), more attention is drawn to disgust stimuli compared to fearful and neutral stimuli (13, 19, 20). Nonetheless, Santos et al. (21) showed in a superimposed face/place task that, when attention was directed away from faces, fear attracted automatic attention, while attention to disgust stimuli was more voluntary, possibly because it is less relevant for avoiding danger. Therefore, Knowles et al. (16) concluded that fear may involve a stronger reliance on automatic bottom-up attentional processes, while disgust processing may involve more voluntary top-down cognitive control. Furthermore, due to the lack of urgency in disgust, the authors argue that there is time and cognitive control to make the voluntary top-down strategic decision to withdraw attention from stimuli (attentional avoidance). However, this is in contrast to the heightened attentional engagement with disgust-evoking stimuli in early processing as described above, whereby this inconsistency in research remains unresolved. One way to measure cognitive control in relation to emotional engagement is through behavioral inhibition. Cognitive control is defined by the processes that allow us to interact with our complex environment in a goal-directed manner (22). Behavioral inhibition is the ability to interrupt an already-initiated reaction (23), and it can be measured using a go/no-go task.

Fear and disgust are strongly linked to different psychiatric disorders such as specific phobias, eating disorders, and the washing sub-type of obsessive–compulsive disorder (C-OCD). Here the disease avoidance system disgust and the defense system (contamination) fear are relevant maintaining factors of C-OCD (3), whereas other motivational systems, e.g., approaching behavior, are less disorder specific. Furthermore, several authors have highlighted the importance of maladaptive cognitive processes as disorder-specific maintenance factors [for a review, see Knowles et al. (9)]. For example, using a go/no-go task, Adams (24) found worsened behavioral inhibition to contamination threat stimuli in disgust-prone individuals, which was assessed across different levels of contamination-based OC symptoms (C-OC symptoms). Moreover, in disgust-prone individuals, attention is allocated faster on the disgust stimulus (15), more disgust is elicited (18), and the stimulus is avoided more strongly (25). Furthermore, many studies, for example, Krug and Carter (26), showed that higher-trait anxiety is associated with impairment (slower response time and decreased accuracy) in high-conflict tasks. These results seem to suggest that disgust-prone individuals show a stronger urgency in response to contamination threat stimuli, which could be—similar to the high-urgency emotion fear—associated with problems in cognitive control. However, because Adams (24) did not differentiate disgust and fear, it remained unclear whether the fearful or/and the disgust aspect of contamination threat contributed to the failure of inhibition. However, this is important to gain a better understanding of the impact of fear and disgust on the development and maintenance of psychiatric disorders and improve tailoring necessary therapeutic interventions. The aim of the present study is therefore to clarify whether behavioral inhibition is given as a characteristic of the low-urgency emotion disgust and thus indicates self-imposed attentional avoidance in comparison to fear. We were further interested in exploring whether more strongly disgust-prone individuals experience higher urgency and therefore more fear-like responses.

In order to test these assumptions, the present study—like Adams (24)—uses a go/no-go task to measure behavioral inhibition (23). Hereby the errors of commission (false alarms during no-go trials) are the main measures of behavioral inhibition, while errors of omission (misses) can be seen as a measure of attention, where more errors of omission reflect more attentional engagement with the preceding stimuli. To date, two studies have investigated disgust-associated stimuli using a go/no-go task: contrary to the findings of Adams (24) described above, Xu et al. (20) found no differences in a healthy population in behavioral inhibition between disgust, fear, and neutral stimuli in the context of a masked go/no-go task, although they masked the emotional stimuli to measure unconscious inhibitory control, which could have reduced the emotional effects. Thus, far, no study has examined the differences between fear and disgust in the context of behavioral inhibition, especially based on the theoretical considerations about the urgency of disgust (16). In light of these findings and theoretical considerations, we would expect at the behavioral level (1.1) a strong emotional engagement and therefore more errors of omission in trials involving fear- and disgust-related stimuli compared to neutral stimuli. However, despite the strong emotional engagement with fear and disgust stimuli, we expect (1.2) that the high urgency of fear results in more errors of commission in trials involving fear-related stimuli compared to disgust-related and neutral stimuli. All expected disgust- and fear-related changes are listed in Table 1 in comparison to the neutral category.

**Table 1:** Expected change compared to the neutral category.

	**Disgust**		**Fear**
Level of C-OC symptoms	Low	High	–
Errors of omission^a^	More errors	More errors	More errors
Errors of commission^b^	No change	More errors	More errors
Heart rate	Decelerated	Stronger decelerated	Accelerated
M. levator labii	Increased	Stronger increased	No change
M. corrugator supercilii	No change	No change	Increased

*C-OC symptoms, contamination-related obsessive–compulsive symptoms*.

a *Measure of attention*.

b *Measure of behavioral inhibition*.

Physiological measures seem to be a plausible way to assess the experienced intensity of disgust and fear independently and more indirectly than direct ratings: the oral, visceral defense reaction of disgust is characterized by nausea (27) and is accompanied by a parasympathetic activation, which is reflected in a reduced heart rate (28). The prototypical facial expression is characterized by facial closure and measured over the m. levator labii. In contrast to disgust, fear is accompanied by a general activation *via* an increased sympathetic activation, which can initiate a rapid combat and flight reaction (29). The prototypical facial expression is characterized by an opening of the mouth and eyes (27) measured over the m. corrugator supercilii. For disgust-prone individuals, Broderick et al. (30), for example, found that subjects with high contamination fear experienced stronger disgust and stronger heart rate deceleration, while there is—to our knowledge—no research on disgust proneness and facial muscle activity yet. At the physiological level, we therefore expect a reduction in the heart rate change during disgust stimuli and an increase during fearful stimuli (2.1). When activating the facial muscles (EMG), we expect an increased activation of the m. levator labii during disgust and an increased activation of the m. corrugator supercilii during fearful stimuli (2.2.).

In disgust-associated mental disorders, disgust may be processed more like fear (high urgency), which appears to occur in vulnerable individuals and affects the development and maintenance of disorders. For disgust-prone individuals (characterized by higher subclinical OC symptoms), we would expect that these effects diminish because disgust is experienced as more threatening. We therefore expect (3) that disgust-prone individuals experience higher urgency and therefore more fear-like responses: less behavioral inhibition (more errors of commission), more attentional engagement (more errors of omission), a stronger reduction in heart rate due to the amplification of the physiological effect, and exploratorily more activity of the m. levator labii in response to disgust stimuli compared to less disgust-prone individuals, while no differentiating effect in response to fearful stimuli is expected.

## Methods

### Participants

We calculated a necessary sample size with G^*^power [V. 3.1; (31)] of 60, assuming a medium effect size (*f* = *0.2*5) derived from the results of Adams (24), a power of 0.95, an α of 0.05, a correlation of 0.3 between the repeated measures (estimated from our previous data and which is, for reference, also the mean correlation in the present study) for a mixed-subject ANOVA with two between-subject groups (PI low vs. PI high) as well as three within-subject emotion conditions (disgust vs. fear vs. neutral) for the dependent variable errors of commission. Note that the independent variable PI is solely split into PI low and PI high to be able to compute the power analysis with G^*^power but will be treated as a linear predictor in the analysis. A total of 58 voluntary subjects participated in this study, of whom four had to be excluded due to severe artifacts in the heart rate data. The exclusion did not change the behavioral results. Therefore, 54 participants (48 female, six male) were included in the following analysis, resulting in a sufficiently powered investigation sample. These 54 participants were, on average, 20.9 years old (SD = 3.9, range: 18–40 years). All participants had graduated from high school (“Abitur”), five participants held a college degree, and all were Bachelor students at the University of Leipzig. They received course credit for their participation. Two participants were not native German speakers, although both were fluent in the German language. All participants also took part in a second experiment, which was randomly performed before or after this experiment and for which we statistically controlled for, although no impact on the dependent variables was found. In this experiment (duration, 30 min), disgust and fear movies were shown, and text statements were rated concerning disgust and fear experience (32). The study was approved by the local ethics committee of the University of Leipzig (329-14-06102014). We report how we determined our sample size, all data exclusion (if any), all manipulations, and all measures in the study (33).

### Measures

The absence of any current psychological disorder was confirmed by the Mini-international Neuropsychiatric Interview based on DSM-IV M.I.N.I. (34), the German version of Ackenheil et al. (35). Three participants had been diagnosed with a previous but remitted psychological disorder before (major depression or bulimia nervosa). No participant was in psychotherapy or on psychopharmacologic treatment during the time of the experiment. Dimensional symptom severity of contamination-based obsessive–compulsive symptoms was assessed with a German translation of the Padua Inventory—Washington State University Revised (36, 37) as a measure of disgust proneness. Depressive symptoms were measured using the second revision of the Beck Depression Inventory [BDI-II; (38)]. In order to measure trait anxiety, the State Trait Anxiety Inventory Trait Scale [STAI-T, (39)] was applied. These two questionnaires were used to differentiate the trait disgust proneness from trait anxiety, i.e., similar valence, and depression, i.e., generally negative mood, respectively.

### Stimuli and Material

As emotional stimuli, 14 fear, 14 disgust, and 14 neutral pictures were used to induce emotional arousal and valence in this study. Among these, 10 fear, seven disgust, and all neutral pictures were selected from the International Affective Picture System^1^ [IAPS; (40)], while four fear-related and seven disgust-related pictures were additionally selected from the Internet. The chosen IAPS pictures had been used in several previous studies [e.g., (41–43)]. All pictures used in the present study were validated in a first pilot study (Appendix A). The fear category contained the pictures with the highest fear rates and low disgust rates, the disgust category contained the pictures with the highest disgust ratings and low fear rates, and the neutral category contained the pictures with the lowest ratings of fear and concurrently the lowest ratings of Visual complexity was assessed through a second pilot study (Appendix B), whereby the three emotion three emotion categories did not significantly differ from each other [*F*_(2, 93)_ = 1.405, *p* = 0.251, BF_10_ = 0.011]. Social information (how many people and what social information is presented in assessed by 11 expert ratings (Appendix C), whereby the fear pictures contained significantly more significantly more social information compared to disgust and neutral pictures [*F*_(2, 27)_ = 13.64, *p* < 0.001, BF_10_ > 1,000]. The luminance of the pictures was calculated by measuring the weighted RGB color space with MATLAB © (Appendix D), whereby the disgust pictures' luminance was significantly increased compared to that of neutral and fear pictures [*F*_(2, 39)_ = 7.685, *p* = 0.002, BF_10_ = 24.35]. Hence, all behavioral and physiological results were controlled for social information and luminance. All pictures were 16 cm in width and 12 cm in height. The response targets were the symbols # and $, which were presented in black on a white-colored 36-cm-wide and 27-cm-high screen. The screen at a distance of 50 cm and responded through a LiTong RTBox reaction time box (44). The MATLAB©-based Psychtoolbox (45, 46) was used to run the experiment on a PC with a 19-in. Iiyama Master Pro 454 CRT monitor (resolution 1,024 × 768 px; refresh rate, 100 Hz). rate, 100 Hz).

### Experimental Design and Procedures

In the beginning, all participants were informed about the voluntariness as well as the procedure and possible inconveniences of the experiment. Written informed consent was obtained from all participants. Subsequently, the participants were asked to work through the M.I.N.I. interview and all other questionnaires. A within-subject design was applied with the within-subject factors emotional category (fear, disgust, and neutral), response (go and no-go), and block (1, 2, 3, and 4). The participants were tested in a small, dimmed, electrically shielded, and sound-attenuated booth. The instructions were read out loud to the participants by the instructor and were given again in written form. On the emotional go/no-go task, task-irrelevant disgust, fear, or neutral pictures were presented before a task-relevant visual stimulus. The participants were instructed to press a key only when the visual go-stimulus occurred (#) and withhold the key press for the no-go stimulus ($). Right-handers were instructed to use their right hand and left-handers their left hand. Furthermore, the participants were instructed to keep their arms and hands still during the experiment. The participants were not informed about the number of practice or experimental blocks and trials for motivational reasons, whereas only the approximate time duration of 40 min was stated. The experiment comprised a practice block with nine neutral pictures. If 66% of the trials in the practice block had been responded to correctly, the participants were allowed to start with the experimental blocks; otherwise, the participants were kindly asked to repeat the practice block. Afterwards, the participants were told to start with the first of four experimental blocks. Each experimental block comprised 42 single trials, which summed up to the total number of 168 trials. Half of the trials were go trials, while the other half comprised no-go trials (47). The participants, pictures, and go or no-go trials were randomly assigned ahead of the experiment. In between the experimental blocks, the participants were allowed to manage the break duration individually and were provided with water and cookies for them to maintain their concentration and to reduce fatigue effects. If breaks of different lengths had an effect, this effect should have affected all conditions equally in the within-subject design. Each practice and experimental trial started with a 500-ms presentation of a black fixation cross in the center of the screen. Following the fixation cross, one of the randomly selected neutral or emotional pictures appeared in the center. After 250 ms, the picture disappeared, and the target symbol was presented at the same position, which elapsed when the participants responded correctly. If the participants responded slower than 400 ms, the feedback “zu langsam” (too slow) appeared on the screen. If the participants responded in a no-go trial, the feedback “falsch” (wrong) appeared on the screen. Afterwards, the pictures were presented again for 8 to 10 s, followed by a 1–3-s inter-trial interval to measure the psychophysiological response to the emotional stimuli (which required longer presentation times). Depending on how long the picture was presented (8–10 s), the difference to 11 s (1–3 s) was used as a physiological baseline before a new trial was started. The procedures and presentation times (figure in Appendix E)—except for the second picture presentation—were applied as suggested by Houwer and Tibboel (47).

In the go/no-go task, two mistakes could occur: first, errors of omissions in go trials (misses), where the responses are omitted or are given too late, whereby such errors are defined as a measure of sustained attentional engagement to the preceding emotional stimulus; and second, errors of commission in no-go trials (false alarms), where the prepotent response cannot be inhibited, which are defined as a measure of (failed) behavioral inhibition (23).

### Psychophysiological Data Recording, Reduction, and Analysis

The physiological data was recorded with Brainproducts BrainAmp MRI EEG amplifiers and the BrainVision Recorder software (Gilching, Germany) throughout the run of the complete experiment at a sampling rate of 500 Hz. Only peripheral physiological activity was recorded in this experiment. For the statistical analyses, only the episodes during the 8 s of the second picture presentation in each trial were used. Six EMG, two ECG, one reference, and one ground electrode were attached to the participant before the experiment started. Two electrodes were placed over the right eyebrows on the m. corrugator supercilii, two electrodes were placed under the nose on the m. levator labii, the reference electrode was placed behind the right ear, and the ground electrode was placed on the center of the forehead (48). The two heart rate electrodes were placed on the left and right inner side of the forearm. All physiological data were pre-processed with the MATLAB©-based EEGlab toolbox (49). The ECG data was filtered using a Hamming-windowed FIR 1-Hz high-pass and 40-Hz low-pass filter (EEGLAB's “eegfiltnew”). R peaks were singled out by the MATLAB©-based toolbox AMRI (50), followed by a visual control. The EMG data was rectified and filtered with a 20–200-Hz band-pass filter. The EMG data was additionally filtered by generating means across a sliding 200-ms window for each data point, performing a moving window average. The EMG and ECG change was calculated by the difference between the picture presentation and baseline, whereby the baseline contained the 1–3 s after the offset of the preceding picture presentation and before the start of this trial.

In order to analyze facial muscle activity, only trials within two standard deviations of facial activity change (51–53) were included (m. corrugator supercilii: 94.42% trials; m. levator labii: 95.71% trials). For the analysis of the heart rate, 90.47% of all data were included (missing data: 4.65% trials; >2 SD: 4.88% trials).

### Statistical Analysis

For the statistical analysis, the software R (54) was applied. The theoretical hypotheses were investigated using either a one-way ANOVA or general linear mixed-effects model analysis (GLM) because multiple observations were collected from each participant^2^. In order to perform the GLM analyses, the R-based package “lme4” [version 1.1-10, (56)] was used. The *p*-values were obtained by likelihood ratio tests, hierarchically testing a more complex against a less complex model. GLM models were created for behavioral and physiological analysis, which comprised fixed-effect variables corresponding to the theoretical approaches tested. Therefore, two random intercepts—participant and picture number—were included in the model as random variables based on the need to use the maximal random effect structure (57). This structure was also not improved by adding the order of the experiments as additional random intercept. The entire forward model selection process is presented in Appendix F. For the best-fitted model, the conditional *R*^2^–as variance explained by the entire model, including both fixed and random effects—is reported in the report section, while marginal *R*^2^ is also reported in Appendix F. *R*^2^ for GLM was calculated in line with Nakagawa and Schielzeth (58) using the R package “MuMIn” (59). In the case of all models reported, other more complex models including and controlling for clinical variables (STAI-T, BDI-II) did not explain more variance. The effect sizes for the one-way ANOVAs with repeated measures were calculated using the R package “rstatix” [version 0.4.0; (60)]. Hereby the generalized eta-squares (η^2^) are reported. A Shapiro Wilk test for normality was calculated for each of the dependent variables. The normality assumption was violated for all dependent variables except for the heart rate change (*W* = 0.986, *p* = 0.119). However, due to the sample size, an approximate asymptotic normal distribution for each of these variables can be assumed (61). A Levene's test for homogeneity of variance was tested for the dependent variables across the emotion categories. For the Levene test, the R package “car” [version 3.0-3; (62)] was used. According to the Levene test, the prerequisite for homogeneity of variance is given, except for the errors of commission [*F*_(2, 159)_ = 3.917, *p* = 0.022]. Because a within-subject design was applied, ANOVA is quite robust to heterogeneity of variance (63). For the within-subject factor time, a Mauchly's test for sphericity was carried out. When the sphericity was violated, the results were corrected by the more conservative Greenhouse–Geisser correction (ε), which is reported in the “RESULTS” section. For multiple comparisons in follow-up *t*-tests, the results were corrected by the Bonferroni procedure. However, this did not change the interpretation of the results in any case. Bayesian *T*-tests and ANOVAs were calculated with the R package “BayesFactor” (64). BF_10_ (Bayes Factor) reports the likelihood ratio of the posterior probability of the alternative model (H_1_) given the data against the posterior probability of the null model (H_0_) given the data. All relevant data are published under https://osf.io/K6AEU/ in the folder: Fink-Lamotte, Widmann et al._Cognitive Processing_Poster and Publication/2020_Data for publication.

## Results

### Behavioral Results

Among all incorrect responses (11.31% of trials), 64.72% were made in go trials (omissions) and 35.28% in the no-go trials (commissions). In order to analyze reaction times, only the correct responses to the go trials and reaction times (RT) longer than 0.1 s were taken into account for the following analysis (98.52% trials). Preceding emotions had no differential effect between the three emotion categories on go trials [*F*_(2, 106)_ = 3.01, *p* = 0.053, BF_10_ = 0.079] nor on no-go trials [*F*_(2, 83)_ =0.81, *p* = 0.447, BF_10_ =0.086]. Therefore, the repeated picture presentation for physiological measurement within one trial had no impact on the behavior results of the subsequent trial. All best-fitted models were controlled for the PI Washing Score (*M* = 3.981, SD = 2.871, range = 0–11), for the BDI-II Score (*M* = 5.648, SD = 4.5, range = 0–17), and for the STAI-T Score (*M* = 16.63, SD = 9.71, range = 5–45).

#### Response Errors

##### Errors of Omission (Go)

**Figure 1:**
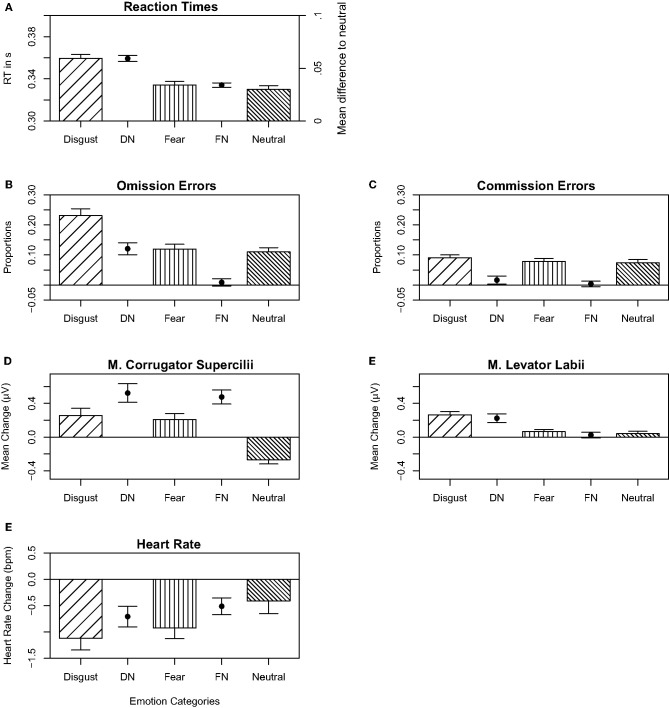
In the upper-left **(A)**, the reaction times are presented; in the second row on the left **(B)** and in the right **(C)**, proportions of the errors of omission and proportions of the errors of commissions are shown, respectively. In the third row in the left **(D)**, the m. corrugator supercilii mean change activity is presented, and in the lower-right **(E)**, the m. levator labii mean change activity is presented. In the lower-left **(F)**, the mean heart rate change is shown. **(A–F)** show data means and standard errors of the disgust, fear, and neutral emotional category. In between the three emotion categories, the mean differences between disgust and neutral as well as between fear and neutral category are presented. When the means differ from the y-axis, the means are shown on the right side of the **(A)**.

**Figure 2:**
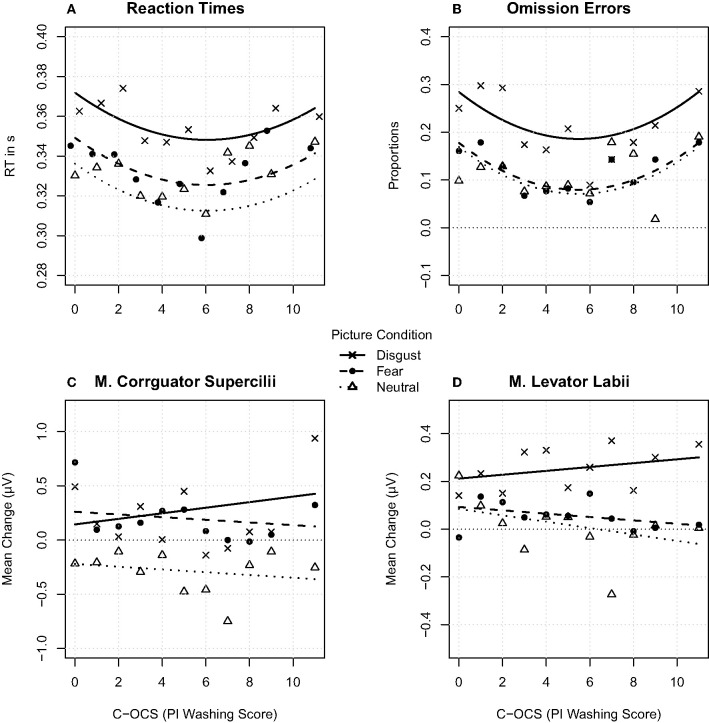
In the upper-left **(A)**, the reaction times are shown; in the upper-right **(B)**, the proportions of the errors of omission are shown; in the lower-left **(C)**, the m. corrugator supercilii mean change activity is presented; and in the lower-right **(D)**, the m. levator labii mean change activity is presented. **(A–D)** show data in relation to the C-OC symptom score to the disgust, fear, and neutral category. The small dots represent the aggregated mean values for each emotion category and each PI Washing Score.

Errors in the go trials (omissions) occurred more often after disgust pictures compared to fear and neutral pictures. A one-way ANOVA resulted in a significant main effect for emotion category [*F*_(2, 114)_ = 36.223, *p* < 0.001, η^2^ = 0.153, BF_10_ > 1,000, ε = 0.76] even after sphericity corrections (Figure 1B). The main effect emotion category was driven by more errors of omission after disgust (*M* = 22.02% during all disgust trials, SD = 2.39) compared to fear [*M* = 11.38%, SD = 1.17, *t*_53_ = 7.042, *p* < 0.001, *d* = 0.736, BF_10_ > 1,000] and neutral pictures [*M* = 10.52%, *SD* = 0.88, *t*_53_ = 6.09, *p* < 0.001, *d* = 0.845, BF_10_ > 1,000]. In a GLM, a model containing two main effects of emotion category and a polynomially adjusted PI Washing Score explained in a tendency more variance (Akaike information criterion, AIC = 3,459.9) compared to a model containing a main effect emotion category [AIC = 3,460.7; $\chi_{\left(2\right)}^{2}$ = 4.779, *p* = 0.09, *R*^2^ = 0.18] (Appendix F; Figure 2B)^3^. The main effect for PI Washing Score can be best described by a U-shaped relationship between more errors for participants with lower PI Washing Scores and participants with higher PI Washing Scores and lower errors of omission for participants with scores in between across all emotion categories.

##### Errors of Commission (No-Go)

Errors in the no-go trials (commissions) did not differ between the emotion categories. A one-way ANOVA resulted in no significant effect for emotion category [*F*_(2, 106)_ = 1.198, *p* = 0.306, η^2^ = 0.01, BF_10_ = 0.18, ε = 0.87], with or without sphericity corrections (Figure 1C). There were not more errors of commission during disgust (*M* = 8.92%, SD = 0.39) compared to fear (*M* = 7.67%, SD = 0.44) and neutral pictures (*M* = 7.34%, SD = 0.51, *p* > 0.2, *d* < 0.233, BF_10_ < 0.33). In a GLM, models containing the single main effect for emotion category (AIC = 2,489.3) or interaction effects for emotion category and PI Washing Score (AIC = 2,494.5) or polynomially adjusted interaction (AIC = 2,794.3) did not explain more variance compared to a model containing only the random effects (AIC = 2,488.3) (Appendix F).

#### Reaction Times

Overall, the RT results are similar compared to the results of the errors of omission (Figure 1A). The one-way ANOVA for the main effect emotion category became significant [*F*_(2, 106)_ = 87.159, *p* < 0.001, η^2^ = 0.195, BF_10_ = 6.29, ε = 0.89) even after sphericity corrections. RTs were longer in the disgust (*M* = 0.356, SD = 0.061) compared to the fear [*M* = 0.332, SD = 0.055, *t*_53_ = 10.936, *p* < 0.001, *d* = 0.911, BF_10_ > 1,000] and the neutral category [*M* = 0.329, SD = 0.054, *t*_53_ = 10.537, *p* < 0.001, *d* = 1.054, BF_10_ > 1,000]. In a GLM, a model containing an interaction effect of emotion category and a polynomially adjusted PI Washing Score explained significantly more variance (AIC = −14,387) compared to a model containing an interaction of emotion category and PI Washing Score [AIC = −14,385; $\chi_{\left(3\right)}^{2}$ = 8.31, *p* = 0.04, *R*^2^ = 0.20] (Appendix F). As presented in Figure 2A, the polynomial regression (*ax*^2^ + *bx* + *c*) fits the data best. This main effect for PI Washing Score can be best described by a U-shaped relationship between longer RTs for participants with lower PI Washing Scores and participants with higher PI Washing Scores and faster RTs for participants with scores in between across all emotion categories.

### Physiological Results

#### EMG Results

##### M. Corrugator Supercilii

The results revealed an increase of m. corrugator supercilii activity in response to pictures of the disgust and fear category and a decrease in the neutral category compared to the baseline. A one-way ANOVA resulted in a significant main effect for emotion category [*F*_(2, 106)_ = 26.441, *p* < 0.001, η^2^ = 0.206, BF_10_ > 1,000, ε = 0.8] even after sphericity corrections (Figure 1D). Muscle activity increased during disgust (*M* = 0.134 μV, SD = 0.933 μV) and fear pictures (*M* = 0.139 μV, SD = 0.879 μV) and decreased during neutral pictures (*M* = −0.195 μV, SD = 0.844 μV) compared to the baseline. The differences between the neutral and the fear category [*t*_53_ = 6.43, *p* < 0.001, *d* = 1.179, BF_10_ > 1,000] as well as the neutral and disgust category became significant [*t*_53_ = −5.376, *p* < 0.001, *d* = 1.13, BF_10_ > 1,000], while the difference between disgust and fear category did not become significant [*t*_53_ = 0.662, *p* = 0.511, *d* = *0.0*78, BF_10_ = 0.192]. In a GLM, a model containing the interaction effect for emotion category and PI Washing Score explained significantly more variance (AIC = 28,768) compared to a model with two main effects emotion category and PI Washing Score [AIC = 28,778; $\chi_{\left(1\right)}^{2}$ = 14.19, *p* < 0.001, *R*^2^ = 0.09] (Appendix F). As presented in Figure 2C, the general linear regression fits the data best. The interaction emotion category × PI Washing Score was driven by an increased corrugator supercilii activity during disgust pictures (bx = 0.006, a0 = 0.027) compared to reduced activity during fear (bx = −0.013, a0 = 0.262) and neutral pictures (bx = −0.014, a0 = −0.219) as the C-OC scores increased.

##### M. Levator Labii

The results revealed a stronger increase of m. levator labii activity after disgust compared to fear and neutral pictures in relation to the baseline. A one-way ANOVA resulted in a significant main effect for emotion category [*F*_(2, 106)_ = 17.944, *p* < 0.001, η^2^ = 0.19, BF_10_ > 1,000, ε = 0.83] even after sphericity corrections (Figure 1E). The main effect was driven by increased facial activity after disgust (*M* = 0.239. μV, SD = 0.72 μV) compared to fear (*M* = 0.072 μV, SD = 0.639 μV) and neutral pictures (*M* = 0.038 μV, SD = 0.672 μV). The differences between the disgust and the fear category [*t*_53_ = 4.722, *p* < 0.001, *d* = 0.922, BF_10_ > 1,000] as well as the disgust and neutral category [*t*_53_ = 4.675, *p* < 0.001, *d* = 0.985, BF_10_ = 961.99] became significant, while the difference between the neutral and fear category did not become significant [*t*_53_ = 0.798, *p* = 0.428, *d* = *0.1*37, BF_10_ = 0.2]. In a GLM, a model containing the interaction effect for emotion category and PI Washing Score explained significantly more variance (AIC = 18,086) compared to the model with two main effects emotion category and PI Washing Score [AIC = 18,092; $\chi_{\left(3\right)}^{2}$ = 12.08, *p* = 0.001, *R*^2^ = 0.04] (Appendix F). As presented in Figure 2D, the general linear regression fits the data best. The interaction emotion category × PI Washing Score was driven by an increased levator labii activity during disgust pictures (bx = 0.008, a0 = 0.023) compared to reduced activity during fear (bx = −0.006, a0 = 0.087) and neutral pictures (bx = −0.016, a0 = 0.103) as the C-OC scores increased.

#### ECG Results

The overall results revealed a decrease in heart rate during disgust and fear pictures and during neutral pictures compared to the baseline. A one-way ANOVA resulted in a significant main effect for emotion category [*F*_(2, 106)_ = 8.986, *p* < 0.001, η^2^ = 0.04, BF_10_ = 94.02] (Figure 1F). Overall, the heart rate decrease, compared to the baseline, was stronger during disgust (*M* = −0.914 bpm, SD = 1.593 bpm) compared to fear (*M* = −0.83 bpm, SD = 1.36 bpm) and neutral pictures (*M* = −0.234 bpm, SD = 1.68 bpm). The differences between the neutral and the fear category [*t*_53_ = 3.85, *p* < 0.001, *d* = 0.379, BF_10_ = 77.72] as well as the neutral and the disgust category [*t*_53_ = 3.769, *p* < 0.001, *d* = 0.415, BF_10_ = 61.53] became significant, while the difference between disgust and fear category did not become significant [*t*_53_ = 0.447, *p* = 0.657, *d* = *0.0*56, BF_10_ = 0.163]. In a GLM, no other model containing main effects or interaction effects with PI Washing Score explained more variance compared to a model containing only a main effect emotion category (AIC = 51,347, *R*^2^ = 0.07) (Appendix F).

## Discussion

The aim of the present study is to clarify whether behavioral inhibition during disgust engagement is characteristic of a low-urgency emotion and thus indicates self-imposed attentional avoidance in comparison to fear. Therefore, a go/no-go task was conducted to investigate the extent to which disgust, as a low-urgency emotion, differs from the high-urgency emotion fear in terms of behavioral inhibition while eliciting high emotional engagement. Contrary to our expectations, at the behavioral level, no differences between the emotion conditions regarding behavioral inhibition could be found, but significant differences regarding emotional engagement were shown. Furthermore, at the physiological level, the emotion conditions significantly differed in facial muscle activity and heart rate change. Individuals with a higher-trait disgust proneness showed faster reactions and higher activity of the m. levator labii. The results show that different trait levels influence attentional engagement but not behavioral inhibition. In the following two sections, we discuss these results and their implications for theory and future research.

### Behavioral and Physiological Support for the Functional Hypothesis of Disgust

Contrary to our two hypotheses at the behavioral level, no difference was found in behavioral inhibition (errors of commission) between disgust, fear, and neutral stimuli (1.1). However, a significantly stronger attentional engagement (errors of omission) to disgust stimuli was shown compared to fear and neutral stimuli (1.2). At the physiological level, the results show—as expected (2.2)—a disgust-specific stronger activity of the m. levator labii compared to the baseline, whereas the effect on the m. corrugator supercilii activity was emotion non-specific. The effect of the m. corrugator supercilii might actually be less emotion specific, as the muscle might also have been active during disgust-specific eye tearing. This would need to be investigated in more detail in future studies. Contrary to our expectations (2.1), heart rate was significantly reduced in both fear and disgust compared to the baseline, which—according to Giuliano et al. (65)—might indicate that more selective attention associated with greater parasympathetic activity is drawn to the emotional stimuli.

In summary, the results suggest that allocation of attention was directed to disgust and fear stimuli, with stronger emotional and increased attentional engagement after disgust compared to fear stimuli. This arousing and allocation effects were not associated—after neither fear nor disgust stimuli—to any problems with behavioral inhibition. Although these results are contrary to our assumptions, in a different way they still coincide with the broader theoretical assumptions of Knowles et al. (16). Here the results show that, in the sense of the functional perspective on attentional avoidance to disgust, strong emotional engagement elicited by the low-urgency disgust stimuli is associated with less demands on cognitive control compared to the high-urgency emotion fear.

However, this argumentation must be followed with caution: it can probably only be drawn since fear engagement was not as strong as disgust engagement. Although the disgust-specific effects mainly corroborate the findings of several previous studies (66–69), it could also be a methodological issue. There are various studies making serious points about the problems and possible solutions with inducing fear (70–72). However, it remains puzzling because disgust and fear stimuli did not differ in our *a priori*-assessed emotional intensity and visual complexity of the picture set (Appendix A). Nonetheless, they differed in the amount of social information (more on fear pictures) and luminance (higher on disgust pictures). However, because the interaction model did not become significant, this effect seems to be emotion unspecific. Moreover, in this study, a fear-specific physiological sympathetic heart rate acceleration was missed. Nonetheless, at the behavioral and physiological levels, the fearful pictures did not elicit the expected reactivity. Another reason could be that mainly women participated in the study, who are generally more sensitive to disgust. Accordingly, in future experiments, it is suggested to explore the extent to which an increased fear experience fulfills the postulated characteristics of the high-urgency emotion and to examine these effects in a more gender-balanced sample. In summary, the results of the present study can be interpreted in line with the theoretical assumptions of Knowles et al. (16), although future studies should more directly study response urgency and avoidance. Therefore, an eye-tracking paradigm would be an interesting approach. Although there is existing research on the topic of disgust processing with eye-tracking (73–75), one study has yet to examine response urgency directly with eye-tracking.

### The Effect of Trait Disgust Proneness on Behavioral Inhibition

In line with our prediction (3) and despite the small variability of the C-OC symptoms in this healthy sample, we found that individuals with higher C-OC symptoms experienced faster RT—as an indicator of behavioral execution—and, to some extent, more errors of omission as an indicator of behavioral engagement—compared to individuals with a middle range of C-OC symptoms (23). Individuals with higher C-OC symptoms also experienced a stronger activity of the m. levator labii and a stronger activity of the m. corrugator supercilii compared to the participants with lower C-OC symptoms. This indicates a stronger overall activation (76) of a disgust response in participants with higher C-OC symptoms. Therefore, these findings can be interpreted as indicators of higher response urgency. However, in contrast to Adams (24), we could not find less behavioral inhibition (more errors of commission) to disgust stimuli compared to individuals with lower C-OC symptoms, which might be the result of the small variability of the C-OC symptoms in this population. The U-shaped relationship between reaction times as well as errors of omission and PI Washing Score could be a result due to less avoidance or stronger interest in these pictures in participants with lower C-OC scores and initial impairment in people with higher C-OC scores. However, the findings are not yet sufficient to argue that the urgency-related differentiating effects between disgust and fear diminish in disgust-prone individuals, which should be the case for future experiments.

### Limitations and Methodological Considerations

We are aware of some limitations of our study. The first such limitation is the gender ratio of 89% women. A more equal gender ratio might control for disgust-specific gender effects, taking into account the notion that women tend to be more sensitive to disgust (77, 78). Nonetheless, the exact same results were found by calculating all results again excluding the seven male subjects, and therefore our results have to be seen as applying primarily to women. Second, as mentioned above, the go and no-go trials were allocated on a 50:50 basis, which was adopted from Houwer and Tibboel (47). A greater percentage of go trials could be relevant to provoke prepotent responses to measure response inhibition. A third limitation of this study is the non-clinical population, although previous research [for reviews, see Abramowitz et al. (79)] have postulated that thoughts and behaviors in OCD differ more in quantitative rather than qualitative aspects from those observed in non-clinical individuals. Therefore, the basic aspects of OCD (e.g., attentional processing) can be investigated on a continuum between non-clinical individuals and OCD patients. Although our healthy sample only represented a small range of OCD symptom severity, the significant effects found in the present study support this methodological approach. A fourth limitation is that the effect of preceding emotions showed a *p*-value of 0.053, which, strictly speaking, shows that there is no significant effect. However, it is not completely out of question that the repeated picture presentation might have had an effect on the results. A fifth limitation is that we only controlled for social information; here it would be interesting in future studies to see if “animacy” is a more relevant control variable. The fact that animates (i.e., living things, such as other people or animals) tend to capture attention in a more automatic manner than inanimates [*i*. *e*., non-living things or objects; (80–83)] should be considered more thoroughly. Another limitation is that disgust and fear images differ strongly in luminance, and thus, even though controlling for it, maybe the effect cannot be distinguished from the emotional effect. Since fear images are often dark, even luminance might be an important factor of emotional reactivity.

### Conclusions and Implications for Further Research

The findings of this multi-method study endorse the main hypothesis, according to which behavioral inhibition of disgust is not impaired by increased behavioral and physiological disgust engagement (in comparison to fear and neutral stimuli). Since generally few commission errors were made, these findings should be seen as first indications. However, this can be carefully interpreted as characteristic of the low-response-urgency of disgust, which is the fundament of the functional perspective on attentional avoidance to disgust (9), even though the results are puzzling given that almost all behavioral and physiological results show an unexpectedly smaller fear than disgust experience. Furthermore, the participants with higher C-OC symptoms showed an increased activation to disgust stimuli, which can be seen as a sign of stronger response urgency to disgusting stimuli in disgust-prone individuals. Nonetheless, behavioral inhibition was not affected by this stronger activation across the different levels of C-OC symptoms. These are the first results to directly test the response urgency to disgust and fear by also broadening the theory in light of disgust-prone individuals. Future studies could more directly manipulate response urgency, strengthen the fear induction, and focus more on the clinical aspects of this theory.

## Data Availability Statement

The raw data supporting the conclusions of this article will be made available by the authors, without undue reservation.

## Ethics Statement

The studies involving human participants were reviewed and approved by Ethics committee of the University of Leipzig. The patients/participants provided their written informed consent to participate in this study.

## Author Contributions

The idea was implemented by JF-L, AW, ES, and CE. The data was evaluated by JF-L, AW, and KS. JF-L, AW, KS, ES, and CE contributed substantively to the preparation of the manuscript. All authors contributed to and have approved the final manuscript.

## Conflict of Interest

The authors declare that the research was conducted in the absence of any commercial or financial relationships that could be construed as a potential conflict of interest.

## Footnotes

^1^The International Affective Picture System and technical manuals (40) are available on CD-ROM and can be obtained on request from the original authors. The IAPS numbers as well as Internet pictures for fear pictures used in this experiment are as follows: 2811, 3500, 6211, 6212, 6300, 6313, 6520, 6550. 6563, 9903, “nightmares 1”, “nightmares 2,” “mutilation 1,” “mutilation 2”; for disgust pictures: 3103, 3170, 3261, 9043, 9301, 9320, 9322, “abscess feed,” “bird 1,” “bird 2,” food, “toilette 1,” “toilette 2,” “serom ear”; for neutral pictures: 2191, 2377, 2411, 2593, 2980, 7006, 7009, 7010, 7035, 7041, 7045, 7052, 7100, 7150.

^2^In a recent article, Aarts et al. (55) simulated the type I error of conventional *vs*. multilevel analyses in such nested designs. The authors found that the α-error increases rapidly when the average number of observations per cluster increases. Furthermore, the α-error was almost doubled when the intercluster correlation increased from 0.1 to 0.5. Because multilevel modeling is not susceptible to the type I error, it was applied in the present study.

^3^The polynomial syntax in R for RTs: lmer(*DV* ~ 1 + *EmoCond* ^*^ poly(*PI*, 2, raw=TRUE) + (1|*Subject*) + (1|*Picture*), data) and for Errors: glmer(*DV* ~ 1 + *EmoCond* ^*^ poly(*PI*, 2, raw=TRUE) + (1|*Subject*) + (1|*Picture*), data, family=binomial).
