# Supplementary material for: Attentional Processing of Disgust and Fear and Its Relationship With Contamination-Based Obsessive–Compulsive Symptoms: Stronger Response Urgency to Disgusting Stimuli in Disgust-Prone Individuals
Source: Front Psychiatry. 2021 Jun 7;12:596557. doi: 10.3389/fpsyt.2021.596557 (PMC8215551; doi:10.3389/fpsyt.2021.596557)
Supplement: Supplementary file 1 [file Data_Sheet_1.docx]

Participants*: N* = 205, 144 female, 59 male, *M_age_* = 26.36, *SD_age_* = 7.54, *R_age_* = 18-84

Method: The pictures were presented in counterbalanced order. After each picture, participants were asked to rate how fearful and how disgusting the picture was on an analog scale from 0 to 100 (0 = not at all disgusting / fearful, 100 = very disgusting/ fearful). The fear category contained the pictures with the highest fear rates and small disgust rates (*M*_F_ = 55.87, *SD*_F_= 11.83, *M*_D_ = 24.02, *SD*_D_ = 10.81), the disgust category contained the pictures with the highest disgust ratings and small fear rates (*M*_F_ = 20.81, *SD*_F_ = 10.9, *M_D_* = 55.0, *SD_D_* = 10.3) and the neutral category contained the pictures with the smallest ratings of fear and concurrently the smallest ratings of disgust (*M*_F_ = 0.7, *SD*_F_ = 0.65, *M*_D_= 1.0, *SD*_D_ = 0.76). To increase the possible choices, we included 20 fear images, 20 disgust images, and 25 neutral images in the preliminary study. Because the IAPS images were not sufficient, 7 images of similar character selected from the Internet were also included to each emotion condition and validated

**Table X. Intensity ratings in the validation study 1, visual complexity in validation study 2, social information in validation study 3 and picture luminance of the selected images**

| Picture category | IAPS /name | description | Valence M (SD) | Arosual  M(SD) | M_Disgust (_*_SD_*_)_ | M_Fear (SD)_ | Diff | Visual Complexity | Social  Information | Luminance |
| --- | --- | --- | --- | --- | --- | --- | --- | --- | --- | --- |
| neutral_1 | 2191 | A man squats on a field | 5.30 (1.62) | 3.61 (2.14) | 0.28 (0.75) | 0.06 (0.24) | 0,22 | 0,20 | 5,27 | 81,95 |
| neutral_2 | 2377 | A woman looks at her cell phone | 5.19 (1.31) | 3.50 (1.95) | 1.00 (3.08) | 2.00 (7.85) | -1,00 | 0,15 | 5,73 | 107,87 |
| neutral_3 | 2411 | A man works on a PC | 5.07 (0.85) | 6.15 (1.87) | 2.05 (4.84) | 1.19 (5.46) | 0,86 | 0,07 | 5,82 | 174,47 |
| Picture category | IAPS /name | description | Valence M (SD) | Arosual  M(SD) | M_Disgust (_*_SD_*_)_ | M_Fear (SD)_ | Diff | Visual Complexity | Social  Information | Luminance |
| neutral_5 | 2593 | People sit in a cafe | 5.80 (1.34) | 3.42 (1.84) | 0.63 (1.45) | 0.13 (0.50) | 0,50 | 0,15 | 5,91 | 76,26 |
| neutral_6 | 2980 | A woman carries potatoes in a basket. | 5.61 (1.50) | 3.09 (1.91) | 2.37 (6.04) | 0.21 (0.71) | 2,16 | 0,06 | 1,27 | 144,53 |
| neutral_8 | 7006 | A white bowl. | 4.88 (0.99) | 2.33 (1.67) | 1.56 (3.47) | 0.20 (0.65) | 1,36 | -0,28 | 1,36 | 78,61 |
| neutral_9 | 7009 | A blue cup. | 4.93 (1.00) | 3.01 (1.97) | 1.86 (4.94) | 0.14 (0.65) | 1,71 | -0,29 | 1,09 | 86,97 |
| neutral_10 | 7010 | A brown basket. | 4.94 (1.07) | 1.76 (1.48) | 0.37 (1.01) | 1.32 (3.90) | -0,95 | -0,28 | 1,36 | 60,29 |
| neutral_11 | 7035 | A glass on a table. | 4.98 (0.96) | 2.66 (1.82) | 0.94 (3.75) | 0.00 (0.00) | 0,94 | 0,01 | 1,27 | 149,22 |
| neutral_12 | 7041 | Brown wooden baskets. | 4.99 (1.12) | 2.60 (1.78) | 0.00 (0.00) | 1.24 (4.30) | -1,24 | -0,18 | 1,00 | 166,90 |
| Picture category | IAPS /name | description | Valence M (SD) | Arosual  M(SD) | M_Disgust (_*_SD_*_)_ | M_Fear_  _(SD)_ | Diff | Visual Complexity | Social  Information | Luminance |
| neutral_13 | 7045 | A zipper. | 4.97 (0.76) | 3.32 (1.96) | 0.39 (1.24) | 1.17 (4.71) | -0,78 | -0,29 | 1,18 | 86,04 |
| neutral_14 | 7052 | Four clothespins. | 5.33 (1.32) | 3.01 (2.02) | 0.00 (0.00) | 1.06 (2.82) | -1,06 | -0,21 | 1,36 | 167,97 |
| neutral_7 | 7100 | A yellow fire hydrant. | 5.24 (1.20) | 2.89 (1.70) | 1.25 (3.51) | 1.00 (2.00) | 0,25 | -0,02 | 1,09 | 116,16 |
| neutral_4 | 7150 | A blue umbrella. | 4.72 (1.00) | 2.61 (1.76) | 1.25 (3.87) | 0.06 (0.25) | 1,19 | -0,15 | 1,45 | 140,04 |
| disgust_1 | 3103 | Legs with a skin eczema. | 2.07 (1.27) | 6.06 (2.30) | 52.27 (29.98) | 36.19 (32.58) | 16,08 | 0,28 | 4,18 | 151,07 |
| disgust_2 | 3170 | A baby with a tumor in her eye. | 1.46 (1.01) | 7.21 (1.99) | 63.48 (29.92) | 36.41 (33.47) | 27,07 | 0,02 | 4,82 | 119,43 |
| disgust_3 | 3261 | A woman with a breast tower | 1.82 (1.34) | 5.75 (2.64) | 75.77 (23.75) | 44.32 (31.43) | 31,45 | 0,20 | 4,18 | 87,42 |
| Picture category | IAPS /name | description | Valence M (SD) | Arosual  M(SD) | M_Disgust (_*_SD_*_)_ | M_Fear_  _(SD)_ | Diff | Visual Complexity | Social  Information | Luminance |
| disgust_4 | 74 | An open wound on the foot | NA | NA | 49.78 (30.19) | 16.61 (25.53) | 33,17 | -0,27 | 3,64 | 114,55 |
| disgust_5 | bird 1 (feeding) | Birds eating vomit. | NA | NA | 44.83 (38.06) | 18.09 (32.57) | 26,74 | -0,35 | 2,45 | 141,16 |
| disgust_6 | 9043 | A toothless face. | 2.52 (1.42) | 5.50 (2.41) | 57.61 (34.17) | 18.21 (23.02) | 39,39 | -0,07 | 4,00 | 156,63 |
| disgust_7 | 9301 | A dirty toilet, full of excrements | 2.26 (1.56) | 5.28 (2.46) | 74.52 (19.77) | 10.67 (22.59) | 63,86 | -0,37 | 2,18 | 131,52 |
| disgust_8 | 9320 | A dirty toilet with maggots | 2.65 (1.92) | 4.93 (2.70) | 52.73 (29.68) | 10.88 (21.34) | 41,85 | -0,18 | 1,64 | 126,16 |
| disgust_9 | abscess feed | Feet with eczema. | NA | NA | 47.25 (32.75) | 21.83 (25.85) | 25,42 | -0,14 | 3,91 | 182,85 |
| disgust_10 | 9322 | Vomit in the sink. | 2.24 (1.24) | 5.73 (2.28) | 52.69 (31.41) | 14.90 (27.40) | 37,79 | 0,12 | 1,55 | 188,75 |
| Picture category | IAPS /name | description | Valence M (SD) | Arosual  M(SD) | M_Disgust (_*_SD_*_)_ | M_Fear_  _(SD)_ | Diff | Visual Complexity | Social  Information | Luminance |
| disgust_11 | toilette 1 | Feces in the toilet | NA | NA | 42.42 (27.33) | 20.63 (23.59) | 21,79 | -0,20 | 3,91 | 108,30 |
| disgust_12 | toilette 2 | A man reaches into the toilet | NA | NA | 54.42 (29.69) | 5.96 (9.42) | 48,46 | -0,02 | 1,64 | 106,33 |
| disgust_13 | terom ear | Ear from which secretion runs. | NA | NA | 57.83 (29.67) | 20.61 (27.66) | 37,22 | -0,08 | 1,18 | 149,13 |
| disgust_14 | bird 2 | A dead bleeding bird. | NA | NA | 44.45 (32.01) | 16.07 (22.53) | 28,38 | 0,43 | 6,09 | 138,34 |
| fear_1 | 2811 | Somebody is shooting at the viewer | 2.17 (1.38) | 6.90 (2.22) | 16.37 (30.83) | 58.22 (31.84) | -41,85 | 0,05 | 4,73 | 95,65 |
| fear_2 | 3500 | A man threatens another with a knife | 2.21 (1.34) | 6.99 (2.19) | 16.30 (26.03) | 59.41 (30.57) | -43,11 | 0,22 | 6,64 | 80,09 |
| fear_3 | 6211 | Two people storm a room with a machine gun | 3.62 (2.07) | 5.90 (2.22) | 18.20 (30.50) | 55.09 (31.05) | -36,89 | -0,18 | 6,09 | 77,06 |
| Picture category | IAPS /name | description | Valence M (SD) | Arosual  M(SD) | M_Disgust (_*_SD_*_)_ | M_Fear_  _(SD)_ | Diff | Visual Complexity | Social  Information | Luminance |
| fear_4 | 6212 | A child is running away from a man shooting | 2.19 (1.49) | 6.01 (2.44) | 35.45 (37.35) | 59.77 (24.94) | -24,32 | 0,11 | 6,55 | 128,13 |
| fear_5 | 6300 | A knife is brandished threateningly | 2.59 (1.66) | 6.61 (1.97) | 24.15 (34.78) | 61.19 (29.47) | -37,04 | 0,12 | 6,91 | 103,70 |
| fear_6 | 6313 | A men threatens a woman with a knife | 1.98 (1.38) | 6.94 (2.23) | 31.57 (33.25) | 70.89 (29.13) | -39,31 | 0,36 | 5,00 | 78,96 |
| fear_7 | 6520 | A man on his knees is threatened. | 1.94 (1.27) | 6.59 (2.08) | 44.27 (36.70) | 63.62 (29.77) | -19,35 | -0,21 | 5,91 | 146,05 |
| fear_8 | 6550 | A man holds a knife to a woman's neck. | 2.73 (2.38) | 7.09 (1.98) | 32.97 (31.31) | 68.09 (30.61) | -35,12 | -0,15 | 5,55 | 49,70 |
| fear_9 | 9903 | A car accident is depicted | 2.36 (1.35) | 5.71 (2.28) | 11.19 (16.91) | 36.19 (28.79) | -25,00 | -0,35 | 4,18 | 120,81 |
| fear_10 | 6563 | A gun is held to the head of a boy | 1.77 (1.23) | 6.85 (2.18) | 25.07 (33.58) | 57.68 (26.18) | -32,61 | 0,04 | 5,82 | 72,73 |
| Picture category | IAPS /name | description | Valence M (SD) | Arosual  M(SD) | M_Disgust (_*_SD_*_)_ | M_Fear_  _(SD)_ | Diff | Visual Complexity | Social  Information | Luminance |
| fear_11 | nightmare (bed) | A girl lies in bed with a monster underneath | NA | NA | 30.83 (31.36) | 49.03 (33.24) | -18,21 | 0,04 | 4,91 | 21,34 |
| fear_12 | nightmares 2 | A creepy girl goes in the cellar | NA | NA | 26.07 (34.97) | 68.37 (33.10) | -42,30 | 0,00 | 6,36 | 71,87 |
| fear_13 | mutilation 1 | A woman in underwear is gagged | NA | NA | 2.42 (8.24) | 32.88 (30.63) | -30,46 | 0,33 | 5,18 | 33,87 |
| fear_14 | mutilation (child) | A child is threatened to be abused. | NA | NA | 21.42 (30.72) | 41.69 (34.94) | -20,27 | 0,37 | 6,00 | 108,19 |
