# Supplementary material for: Attentional Processing of Disgust and Fear and Its Relationship With Contamination-Based Obsessive–Compulsive Symptoms: Stronger Response Urgency to Disgusting Stimuli in Disgust-Prone Individuals
Source: Front Psychiatry. 2021 Jun 7;12:596557. doi: 10.3389/fpsyt.2021.596557 (PMC8215551; doi:10.3389/fpsyt.2021.596557)
Supplement: Supplementary file 2 [file Data_Sheet_2.docx]

Participants*: N* = 96, female= 61 (63.54%), male = 33 (34.38%), other = 2 (2.08%), *M_age_* = 27.88, *SD_age_* = 5.51, *R_age_* = 20-62

Method: Two pictures were presented at the same time. Picture A was the reference picture, which was the same picture within one participant and was therefore presented in each trial. Picture A changed between the participants and was randomly assigned. Picture B changed and included the other 41 pictures used in this study. Pre study 2 contained 41 trials. Picture position was also randomized. Participants were asked to rate on a 7-point Likert-scale (+3 = A is visual more complex compared to B; -3 = B is visual more complex compared to A) which picture was visual more complex. Before the experiment high visual complexity was defined through different colors, many details and ambiguous information. Whereas low visual complexity was defined through few colors and details as well as precise information. *(“Ein visuell komplexes Bild kennzeichnet sich dabei durch unterschiedliche Farben, viele Details und mehrdeutige Informationen. Ein visuell wenig komplexes Bild durch wenige Farben, wenige Details und klare, präzise Information.”)*

Results:

| Emotion | *M* (visual complexity) | *SD* |
| --- | --- | --- |
| Disgust | -0.04111498 | 2.031580 |
| Fear | 0.04212860 | 2.000214 |
| Neutral | -0.06794425 | 2.085091 |

Scale scores: +3 = A is visual more complex compared to B; -3 = B is visual more complex compared to A

One between-factor ANOVA Emotion: *F*(2,93) = 1.405, *p* = 0.251, ns
