# Supplementary material for: Attentional Processing of Disgust and Fear and Its Relationship With Contamination-Based Obsessive–Compulsive Symptoms: Stronger Response Urgency to Disgusting Stimuli in Disgust-Prone Individuals
Source: Front Psychiatry. 2021 Jun 7;12:596557. doi: 10.3389/fpsyt.2021.596557 (PMC8215551; doi:10.3389/fpsyt.2021.596557)
Supplement: Supplementary file 3 [file Data_Sheet_3.docx]

Participants: expert ratings (MSc in psychology); *N*= 11*;* female= 9 (81.8%), male = 2 (18.18%), *M_age_* = 30.45, *SD_age_* = 3.75, *R_age_* = 25-36

Method: One picture was presented in pre study 3 in each trial. All 42 pictures were presented in counterbalanced order. Participants were considered as experts, by at least holding a Master Degree in Psychology. Before the experiment the degrees of social information (few and many) were defined by examples (below). The participants were asked to rate the degree of social information presented on the pictures on a 7-point Likert scale (1 = No social information; 7 = Many social information).

| Example for less or no social information  *No people are on the picture or only one person, though there is no information about the gender, the situation, a possible social interaction, what the person is planning to do or what the person is thinking or feeling.*  Beispiel für wenig bis gar keine soziale Information: | Example for many social information  One Person or many people are presented on the picture and there is precise information about the gender, the situation, social interaction, what the person is planning to do or what the person is think or feeling.  Beispiel für sehr viel soziale Information: |
| --- | --- |
| *Kein Mensch ist auf dem Bild oder ein Mensch ist auf dem Bild, es ist aber nicht bestimmbar, welches Geschlecht er hat, in welcher Situation er sich gerade befindet, ob er Kontakt zu anderen Menschen hat, was er als nächstes wahrscheinlich vorhat oder was er denkt oder fühlt.* | *Ein Mensch oder mehrere Menschen sind auf dem Bild, es ist bestimmbar, welches Geschlecht er hat, in welcher Situation sich der Mensch gerade befindet, welchen Kontakt er zu anderen Menschen hat, was er als nächstes wahrscheinlich vorhat oder was er denkt oder fühlt.* |

Results:

| Emotion | *M* (Social Information) | *SD* |
| --- | --- | --- |
| Disgust | 3.240260 | 1.950549 |
| Fear | 5.701299 | 1.353426 |
| Neutral | 2.512987 | 2.118197 |

Scale scores: 1 = No social information; 7 = Many social information

One within-factor ANOVA Emotion: *F*(2,27) = 13.64, *p* < 0.001, ***
