# Supplementary material for: Attentional Processing of Disgust and Fear and Its Relationship With Contamination-Based Obsessive–Compulsive Symptoms: Stronger Response Urgency to Disgusting Stimuli in Disgust-Prone Individuals
Source: Front Psychiatry. 2021 Jun 7;12:596557. doi: 10.3389/fpsyt.2021.596557 (PMC8215551; doi:10.3389/fpsyt.2021.596557)
Supplement: Supplementary file 4 [file Data_Sheet_4.docx]

Method: Luminance was calculated using MATLAB © and the standard equation for certain color space: 0.2126*R + 0.7152*G+0.0722*B (Stokes, Anderson, Chandrasekar, and Motta, "A Standard Default Color Space for the Internet - sRGB").

Results:

| Emotion | *M* (lumination) | *SD* |
| --- | --- | --- |
| Disgust | 135.83143 | 28.67990 |
| Fear | 84.86839 | 35.33378 |
| Neutral | 116.94981 | 39.45899 |
