# Supplementary material for: Attentional Processing of Disgust and Fear and Its Relationship With Contamination-Based Obsessive–Compulsive Symptoms: Stronger Response Urgency to Disgusting Stimuli in Disgust-Prone Individuals
Source: Front Psychiatry. 2021 Jun 7;12:596557. doi: 10.3389/fpsyt.2021.596557 (PMC8215551; doi:10.3389/fpsyt.2021.596557)
Supplement: Supplementary file 5 [file Data_Sheet_5.docx]

| + |  |
| --- | --- |


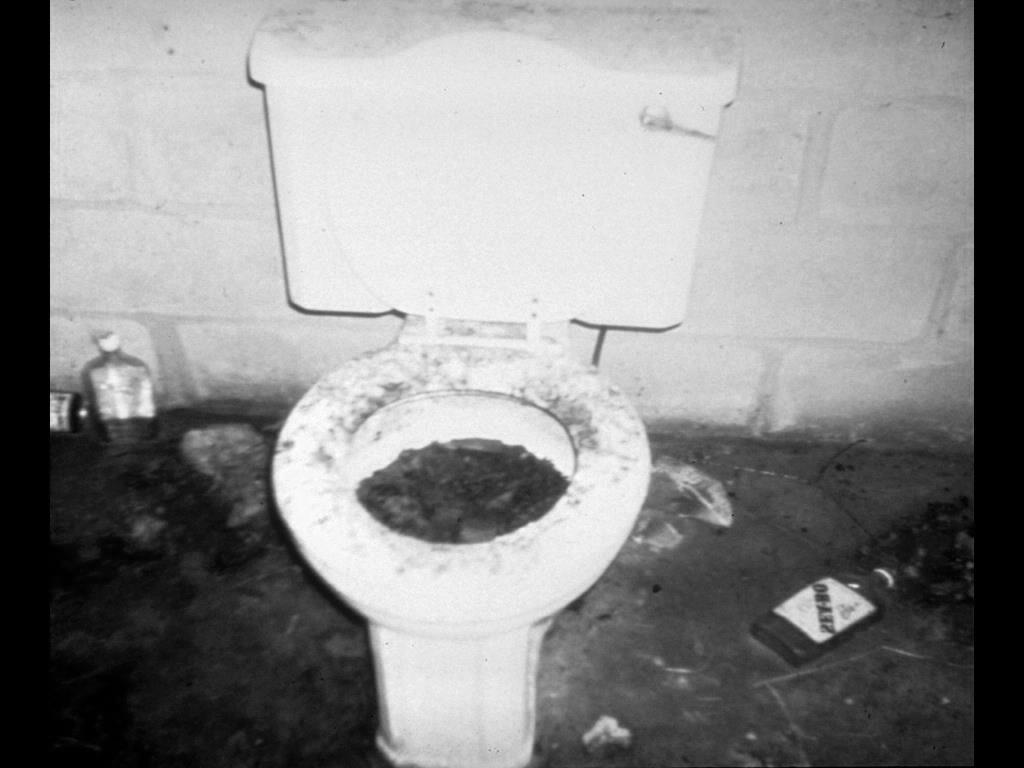


go vs. no-go
 # or $


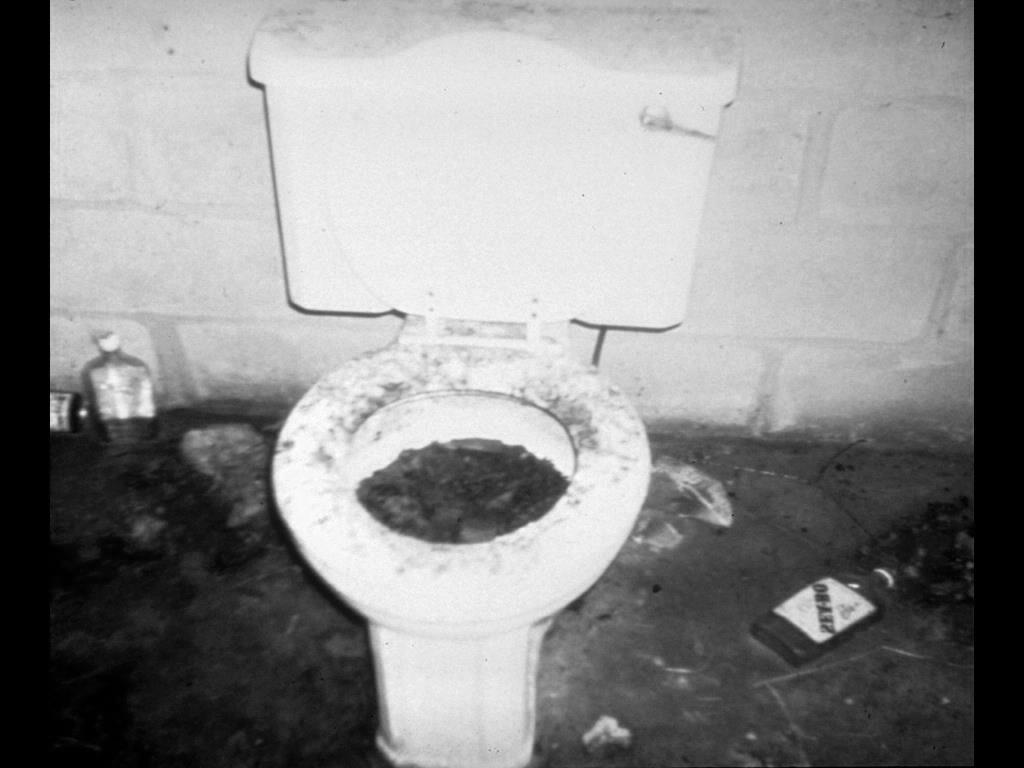


| pause |  |
| --- | --- |

250 ms

650 ms

400 ms

8000 ms

| + |  |
| --- | --- |

The longer second picture presentation was included to assess physiology

*Figure E.* The procedure of one trial.
