# Supplementary material for: Attentional Processing of Disgust and Fear and Its Relationship With Contamination-Based Obsessive–Compulsive Symptoms: Stronger Response Urgency to Disgusting Stimuli in Disgust-Prone Individuals
Source: Front Psychiatry. 2021 Jun 7;12:596557. doi: 10.3389/fpsyt.2021.596557 (PMC8215551; doi:10.3389/fpsyt.2021.596557)
Supplement: Supplementary file 6 [file Data_Sheet_6.docx]

## Appendix F: Linear Mixed Models

### Reaction Times

**Table 6.1.1a. Reaction Times: Forward model selection. (R syntax see 6.2)**

| Model | AIC | deviance | χ^2^(df) | p |
| --- | --- | --- | --- | --- |
| RT ~ 1 + random | -14156 | -14164 |  |  |
| ***RT ~ 1 + Emo*** | ***-14383*** | ***-14395*** | ***χ^2^(2) =230.83*** | ***<.01*** |
| RT ~ 1+ Emo + PI | -14381 | -14395 | χ^2^(1) = .15 | .69 |
| RT ~ 1 + Emo | -14383 | -14395 |  |  |
| RT ~ 1 + Emo * PI | -14385 | -14403 | χ^2^(3) = 7.65 | .05 |
| RT ~ 1 + Emo | -14383 | -14395 |  |  |
| RT ~ 1 + Emo + poly(PI, 2) | -14384 | -14400 | χ^2^(1) = 5.11 | .08 |
| **RT ~ 1 + Emo * poly(PI, 2)** | **-14387** | **-14411** | **χ^2^(3) = 10.85** | **.03** |
| RT ~ 1 + Emo * PI | -14385 | -14403 |  |  |
| **RT ~ 1 + Emo * poly(PI, 2)** | **-14387** | **-14411** | **χ^2^(3) = 8.31** | **.04** |

**Table 5.1.1b. Random effects of the** best fitted model: **RT ~ 1 + Emo * poly(PI, 2)**

| Groups | Variance | Std.Dev. |
| --- | --- | --- |
| Subject | .0005 | .022 |
| Picture Number | .00003 | .006 |
| Residuals | .0028 | .054 |

**Table 5.1.1c. Fixed effects of the** best fitted model: **RT ~ 1 + Emo * poly(PI, 2)**

|  | Estimate | *t*- value | *p* |
| --- | --- | --- | --- |
| (Intercept) | .367 | 46.34 | <.01 |
| EmoCondfear | -.021 | -4.79 | <.01 |
| EmoCondneutral | -.035 | -7.78 | <.01 |
| poly(PI, 2, raw = TRUE)1 | -.007 | -2.14 | .04 |
| poly(PI, 2, raw = TRUE)2 | .001 | 1.86 | .07 |
| EmoCondfear:poly(PI, 2, raw = TRUE)1 | -.002 | -.9 | .36 |
| EmoCondneutral:poly(PI, 2, raw = TRUE)1 | .002 | 1.15 | .25 |
| EmoCondfear:poly(PI, 2, raw = TRUE)2 | .001 | 1.41 | .16 |
| EmoCondneutral:poly(PI, 2, raw = TRUE)2 | .001 | -.29 | .77 |

*R^2^* (marginal) = .054

*R^2^* (conditional) = .197

### Errors of Omission

**Table 5.1.2a. Omission Errors: GLMER-Forward model selection (binomial) (R syntax: 6.3)**

| Model | AIC | deviance | χ^2^(df) | p |
| --- | --- | --- | --- | --- |
| Omissions ~ 1 + random | 3560.7 | 3554.7 |  |  |
| ***Omissions ~ 1 + Emo*** | ***3460.7*** | ***3450.7*** | ***χ^2^(2) = 104.02*** | ***<.01*** |
| Omissions ~ 1 + Emo + PI | 3462.3 | 3450.3 | χ^2^(1) = .407 | .52 |
| Omissions ~ 1 + Emo * PI | 3463.4 | 3447.4 | χ^2^(2) = 2.849 | .24 |
| Omissions ~ 1 + Emo | 3460.7 | 3450.7 |  |  |
| **Omissions ~ 1 + Emo + poly(PI, 2)** | **3459.9** | **3445.9** | **χ^2^(2) = 4.779** | **.09** |
| Omissions ~ 1 + Emo * poly(PI, 2) | 3462.3 | 3440.3 | χ^2^(4) = 5.595 | .23 |

**Table 5.1.2b. Random effects of the** best fitted model: **Omissions ~ 1 + Emo + poly(PI, 2)**

| Groups | Variance | Std.Dev. |
| --- | --- | --- |
| Subject | .555 | .745 |
| Picture Number | .064 | .254 |

**Table 5.1.2c. Fixed effects of the** best fitted model: **Omissions ~ 1 + Emo + poly(PI, 2)**

|  | Estimate | z- value | *p* |
| --- | --- | --- | --- |
| (Intercept) | -.932 | -3.367 | <.01 |
| EmoCondfear | -.868 | -8.180 | <.01 |
| EmoCondneutral | -.964 | -8.889 | <.01 |
| poly(PI, 2, raw = TRUE)1 | -.271 | -2.243 | .0 |
| poly(PI, 2, raw = TRUE)2 | .024 | 2.145 | .03 |

*R^2^* (marginal) = .03

*R^2^* (conditional) = .11

### Errors of Commission

**Table 5.1.3a. Commission Errors: GLMER-Forward model selection (binomial).**

| Model | AIC | deviance | χ^2^(df) | p |
| --- | --- | --- | --- | --- |
| **Commissions ~ 1 + random** | **2488.3** | **2482.3** |  |  |
| Commissions ~ 1 + Emo | 2489.3 | 2479.3 | χ^2^(2) = 2.92 | .23 |
| Commissions ~ 1 + Emo + PI | 2491.3 | 2479.3 | χ^2^(1) = .04 | .85 |
| Commissions ~ 1 + Emo * PI | 2494.5 | 2478.5 | χ^2^(2) = .78 | .68 |
| Commissions ~ 1 + random | 2488.3 | 2482.3 |  |  |
| Commissions ~ 1 + Emo + poly(PI, 2) | 2813.9 | 2799.9 | χ^2^(2) = 2.69 | .26 |
| Commissions ~ 1 + Emo * poly(PI, 2) | 2794.3 | 2794.3 | χ^2^(6) = 6.66 | .16 |

**Table 5.1.3b. Random effects of the** best fitted model: **Commissions ~ 1 + random**

| Groups | Variance | Std.Dev. |
| --- | --- | --- |
| Subject | .291 | .539 |
| Picture Number | .037 | .193 |

**Table 5.1.3c. Fixed effects of the** best fitted model: **Commissions ~ 1 + random**

|  | Estimate | z- value | *p* |
| --- | --- | --- | --- |
| (Intercept) | -2.579 | -23.58 | <.01 |

*R^2^* (marginal) = 0

*R^2^* (conditional) = .025

### M. corrugator supercilii

**Table 5.1.4a. M. corrugator supercilii change (CSC): Forward model selection.**

| Model | AIC | deviance | χ^2^(df) | p |
| --- | --- | --- | --- | --- |
| CSC ~ 1 + random | 29072 | 29064 |  |  |
| ***CSC ~ 1 + Emo*** | ***28776*** | ***28764*** | ***χ^2^(2) = 299.13*** | ***< .01*** |
| CSC ~ 1 + Emo + PI | 28778 | 28764 | χ^2^(2) < .01 | .99 |
| ***CSC ~ 1 + Emo * PI*** | ***28768*** | ***28750*** | ***χ^2^(1) = 14.19*** | ***< .01*** |
| CSC ~ 1 + Emo | 28776 | 28764 |  |  |
| CSC ~ 1 + Emo + poly(PI, 2) | 28778 | 28762 | χ^2^(2) = 2.71 | .10 |
| ***CSC ~ 1 + Emo * poly(PI, 2)*** | ***28768*** | ***28744*** | ***χ^2^(1) = 17.86*** | ***< .01*** |
| **CSC ~ 1 + Emo * PI** | **28768** | **28750** |  |  |
| CSC ~ 1 + Emo * poly(PI, 2) | 28768 | 28744 | χ^2^(3) = 6.38 | .11 |

**Table 5.1.4b. Random effects of the** best fitted model: **CSC ~ 1 + PI * Emo**

| Groups | Variance | Std.Dev. |
| --- | --- | --- |
| Subject | .099 | .314 |
| Picture Number | .007 | .086 |
| Residuals | 1.59 | 1.26 |

**Table 5.1.4c. Fixed effects of the** best fitted model: **CSC ~ 1 + PI * Emo**

|  | Estimate | *t*- value | *p* |
| --- | --- | --- | --- |
| (Intercept) | .144 | 1.65 | .102 |
| PI | .026 | 1.5 | .138 |
| EmoCondfear | .116 | 2.028 | .042 |
| EmoCondneutral | -.365 | -6.342 | <.01 |
| PI:EmoCondfear | -.037 | -3.258 | <.01 |
| PI:EmoCondneutral | -.038 | -3.258 | <.01 |

*R^2^* (marginal) = .033

*R^2^* (conditional) = .094

### M. levator labii change

**Table 5.1.5a. M. levator labii change (LLC): Forward model selection.**

| Model | AIC | deviance | χ^2^(df) | p |
| --- | --- | --- | --- | --- |
| LLC ~ 1 + random | 18248 | 18085 |  |  |
| LLC ~ 1 + Emo | 18092 | 18080 | χ^2^(2) = 159.68 | < .01 |
| LLC ~ 1 + Emo + PI | 18092 | 18080 | χ^2^(1) = .67 | .41 |
| ***LLC ~ 1 + Emo * PI*** | ***18086*** | ***18068*** | ***χ^2^(2) = 12.08*** | ***< .01*** |
| LLC ~ 1 + Emo | 18092 | 18080 |  |  |
| LLC ~ 1 + Emo + poly(PI, 2) | 18095 | 18079 | χ^2^(2) = .41 | .52 |
| ***LLC ~ 1 + Emo * poly(PI, 2)*** | ***18085*** | ***18061*** | ***χ^2^(4) = 18.68*** | ***< .01*** |
| **LLC ~ 1 + Emo * PI** | **18086** | **18068** |  |  |
| LLC ~ 1 + Emo * poly(PI, 2) | 18085 | 18061 | χ^2^(3) = 7.022 | .10 |

**Table 5.1.5b. Random effects of the** best fitted model: **LLC ~ 1 + PI * Emo**

| Groups | Variance | Std.Dev. |
| --- | --- | --- |
| Subject | .009 | .096 |
| Picture Number | .0001 | .01 |
| Residuals | .467 | .683 |

**Table 5.1.5c. Fixed effects of the** best fitted model: **LLC ~ 1 + PI * Emo**

|  | Estimate | *t*- value | *p* |
| --- | --- | --- | --- |
| (Intercept) | .212 | 6.687 | <.01 |
| EmoCondfear | -.119 | -3.829 | <.01 |
| EmoCondneutral | -.127 | -4.088 | <.01 |
| PI | .008 | 1.256 | .21 |
| EmoCondfear:PI | -.015 | -2.390 | .01 |
| EmoCondneutral:PI | .021 | -3.387 | <.01 |

*R^2^* (marginal) = .019

*R^2^* (conditional) = .039

### Heart rate change

**Table 5.1.6a. Heart rate change (HRC): Forward model selection**

| Model | AIC | deviance | χ^2^(df) | p |
| --- | --- | --- | --- | --- |
| HRC ~ 1 + random | 49190 | 49182 |  |  |
| **HRC ~ 1 + Emo** | **49157** | **49145** | **χ^2^(2) = 36.93** | **< .01** |
| HRC ~ 1 + Emo + PI | 49159 | 49145 | χ^2^(1) = .04 | .84 |
| HRC ~ 1 + Emo * PI | 49162 | 49144 | χ^2^(2) = .76 | .68 |
| HRC ~ 1 + Emo | 49157 | 49145 | χ^2^(2) = 36.93 | < .01 |
| HRC ~ 1 + Emo + poly(PI, 2) | 49160 | 49144 | χ^2^(1) = .88 | .65 |
| HRC ~ 1 + Emo * poly(PI, 2) | 49163 | 49139 | χ^2^(3) = 4.47 | .35 |

**Table 5.1.2b. Random effects of the** best fitted model: **HRC ~ 1 + Emo**

| Groups | Variance | Std.Dev. |
| --- | --- | --- |
| Subject | 1.6053 | 1.267 |
| Picture Number | .017 | .134 |
| Residuals | 22.965 | 4.79 |

**Table 5.1.2c. Fixed effects of the** best fitted model: **HRC ~ 1 + Emo**

|  | Estimate | *t*- value | *p* |
| --- | --- | --- | --- |
| (Intercept) | -.962 | -4.849 | <.01 |
| EmoCondfear | .147 | 1.137 | .256 |
| EmoCondneutral | .744 | 5.749 | <.01 |

*R^2^* (marginal) = .004

*R^2^* (conditional) = .069

## Example R SYNTAX FOR LMER SELECTION (Reaction Times):

tmp2 <- dat

lme.pi0 <- lmer(RT ~ 1 + (1| Subj)+ (1| Picture), tmp2, REML=T)

lme.pi1 <- lmer(RT ~ 1 + EmoCond + (1| Subj)+ (1| Picture), tmp2, REML=T)

lme.pi2 <- lmer(RT ~ 1 + EmoCond + PI + (1| Subj)+ (1| Picture), tmp2, REML=T)

lme.pi3 <- lmer(RT ~ 1 + EmoCond + PI + EmoCond + poly(PI, 2, raw=TRUE) + (1| Subj)+ (1| Picture),tmp2, REML=T) #*

lme.pi4 <- lmer(RT ~ 1 + EmoCond + PI + EmoCond * PI + (1| Subj)+ (1| Picture), tmp2, REML=T)

lme.pi5 <- lmer(RT ~ 1 + EmoCond + PI + EmoCond * poly(PI, 2, raw=TRUE) + (1| Subj)+ (1| Picture),tmp2, REML=T) #*

anova(lme.pi0,lme.pi1,lme.pi2,lme.pi4)

anova(lme.pi1,lme.pi3,lme.pi5)

## Example R SYNTAX FOR LMER SELECTION (Omission Errors):

tmp2<- aggregate(resp ~ EmoCond + PI + Block + Picture + Subj, dat[dat$GoCond == "go",], length)

tmp2$wrong <- aggregate(resp ~ EmoCond + PI + Block + Picture + Subj, dat[dat$GoCond == "go",], function(x) {sum(x == "wrong")})$resp

lme.pi0 <- glmer(wrong ~ 1 + (1|Subj)+ (1|Picture), tmp2, family=binomial)

lme.pi1 <- glmer(wrong ~ 1 + EmoCond + (1| Subj)+ (1| Picture), tmp2, family=binomial)

lme.pi2 <- glmer(wrong ~ 1 + EmoCond + PI + (1|Subj) + (1| Picture), tmp2, family=binomial)

lme.pi3 <- glmer(wrong ~ 1 + EmoCond + poly(PI, 2, raw=TRUE) + (1| Subj)+ (1| Picture), tmp2, family=binomial)

lme.pi4 <- glmer(wrong ~ 1 + EmoCond * PI + (1| Subj) + (1| Picture), tmp2, family=binomial)

lme.pi5 <- glmer(wrong ~ 1 + EmoCond * poly(PI, 2, raw=TRUE) + (1| Subj)+ (1| Picture),tmp2, family=binomial)

anova(lme.pi0,lme.pi1,lme.pi2,lme.pi4)

anova(lme.pi1,lme.pi3,lme.pi5)
